# The luxury effect beyond cities: bats respond to socioeconomic variation across landscapes

**DOI:** 10.1186/s12898-019-0262-8

**Published:** 2019-11-01

**Authors:** Han Li, Kevin A. Parker, Matina C. Kalcounis-Rueppell

**Affiliations:** 1 Department of Biology, University of North Carolina Greensboro, Greensboro, NC USA; 2 Present Address: Faculty of Science and Biological Science, University of Alberta, Edmonton, Canada

**Keywords:** Luxury effect, Bats, Land cover, Income, Broad scale, Mobile survey

## Abstract

**Background:**

The luxury effect describes the positive relationship between affluence and organism diversity or activity in urban ecosystems. Driven by human activities, the luxury effect can potentially be found at a broader scale across different landscapes. Previously, the luxury effect relationship has been established within a city for two bat species, the red bat (*Lasiurus borealis*) and the evening bat (*Nycticeius humeralis*). We examined landscape-scale patterns of bat activity distribution—using empirical data for seven bat species for the luxury effect. We also identified bat-land cover associations for each species. Across North Carolina, USA, we used the mobile transect survey protocol of the North American Monitoring Program to record bat activity at 43 sites from 2015 to 2018. We collected land cover and income data at our transect locations to construct generalized linear mixed models to identify bat-land cover and bat-income relationships.

**Results:**

We found that across landscapes, activity of the red bat and the evening bat was positively correlated to income independent of land cover, consistent with previous single-city results. We found a negative relationship between hoary bat (*Lasiurus cinereus*) activity and income. All seven species had specific land cover associations. Additionally, we found a positive interaction term between income and evergreen forest for the red bat and a positive interaction term between income and woody wetland for hoary bat.

**Conclusions:**

Our results demonstrated that the luxury effect is an ecological pattern that can be found at a broad spatial scale across different landscapes. We highlight the need for multi-scale ecology studies to identify the mechanism(s) underlying the luxury effect and that the luxury effect could cause inequity in how people receive the ecosystem services provided by bats.

## Background

The term “luxury effect” describes a functional relationship that links affluence and biodiversity in urban ecosystems [1]. Hope et al. [1] suggested that in urban ecosystems the traditional resource-diversity relationship has been modified by human activities. Instead of natural resources being limiting factors, financial resources that interact with land use, legacy effects, and other sociocultural factors shape the biodiversity patterns in urban areas [1]. The luxury effect has been demonstrated worldwide across different plant and animal species in many urban areas (e.g. [2–6]).

There are several factors that could potentially drive the luxury effect in an urban area (see [7] for a comprehensive review). Affluence, generally measured by median household income, predicts where people live and how people manage their property. These human activities directly affect the physical structure, microclimate, and vegetation of different parts of an urban area [8–11], which can consequently impact animal distributions [12–14]. Human activities are also influenced by the existing environmental heterogeneity and inequity in an urban area and may amplify the heterogeneity and inequity over time [15–18]. Therefore, the core driver of the luxury effect is the difference in human activities caused by the socioeconomic differences.

The luxury effect is found in cities likely because urban ecosystems have the highest human density and are highly impacted by human activities [19]. As the world is urbanizing, it is important to examine whether the luxury effect of urban ecosystems could be found in other landscapes and whether it is evident at a broader scale. At the global scale, studies have shown that terrestrial biodiversity hotspots are in low-income developing countries [20–22]. However, many biodiversity hotspots such as the Amazon rainforest or the south-central region of China have very limited human presence and still contain primary vegetation [20]. In contrast, there has been no empirical study to examine the luxury effect in landscapes that all have been heavily modified by humans and compare the magnitude of this effect across landscapes.

Differences in human activities caused by socioeconomic differences can be found in landscapes other than the urban landscape. In the US, the socioeconomic background of farmers influences whether they would participate in conservation-compatible practices and programs on farmlands [23]. In Australian agroecosystems, whether farmers participate in conservation schemes for farming practices is influenced by complex social factors [24]. Piemonti et al. [25] demonstrated that watershed conservation and management actions could be influenced by landowners’ socioeconomic background. Even in protected areas, various land use activities are driven by socioeconomic factors [26], such as exurban housing developments in areas close to nature for the wealthy [27, 28] or cattle grazing on mesic habitats driven by rent economics [29]. Therefore, it is reasonable to expect that the luxury effect in urban ecosystems can scale up spatially and be found across landscapes.

Previously, a single-city study of bat distribution patterns indicated bats responded to urban affluence variations. Li and Wilkins [4] found that in a medium-sized city (Waco, Texas, USA), two tree-roosting bat species, the eastern red bat (*Lasiurus borealis*) and the evening bat (*Nycticeius humeralis*) had a higher probability of presence in neighborhoods with higher median household income. Further, in this study these two species responded to vegetation coverage, which was consistent with their habitat preference [30]. However, contradictory to studies that examine urban vegetation and affluence (e.g. [8, 31]), vegetation coverage was not correlated to median household income in Waco. The independence of vegetation coverage and median household income, and that both factors affect bat distribution [4], suggests that the luxury effect is more complicated than a simple altering of habitat availability [1].

Bats are able to explore large areas and are associated with different land cover types [32, 33]. In France, a landscape scale study showed that four bat species avoid intensive agriculture whereas the effect of urban land cover varies by species [34]. In the USA, two bat species positively respond to urban land cover [35]. In Italy, the lesser horseshoe bat (*Rhinolophus hipposideros*) distribution at the landscape scale is associated broad-leaved forests [36]. In addition to land cover types, socioeconomic differences among landscapes elements could potentially be perceived by bats and affect distribution.

The objective of our study was to examine if the luxury effect could be found in bats at the landscape scale using empirical data collected across multiple urban centers. We also aimed to identify species-specific bat-land cover associations and test whether the luxury effect and bat-landcover association were two independent processes. Because there are only a few bat species in most parts of the US and the occurrence of these species is common, alpha diversity is less informative than the activity of bats [7]. Hence, we selected seven common North American bat species as the target to examine the luxury effect in species-specific bat activity. The target species were the big brown (*Eptesicus fuscus*), eastern red (*L. borealis*), hoary (*L. cinereus*), silver haired (*Lasionycteris noctivagans*), evening (*Nycticeius humeralis*), tricolored (*Perimyotis subflavus*), and Mexican free-tailed (*Tadarida brasiliensis*) bat. Specifically, we hypothesized that across different landscapes, the luxury effect would be found in *L. borealis* and *N. humeralis* but not in other species based on previous single-city results [4]. We predicted that both species would positively respond to affluence as measured by median household income. Furthermore, we expected no correlation between affluence and any specific type of land cover and species-specific bat-land cover associations.

## Results

Between 2015 and 2018, we collected 10,899 bat passes that met identification criteria. From these bat passes we identified 805 passes of *E. fuscus*, 2609 passes of *L. borealis*, 158 passes of *L. cinereus*, 585 passes of *L. noctivagans*, 1857 passes of *N. humeralis*, 1016 passes of *P. subflavus*, and 350 passes of *T. brasiliensis*. Among the 43 sites, deciduous forest was the most common land cover type followed by evergreen forest and pasture/hay (Table 1). Of all 15 land cover types, 13 were present in all sites (Table 1). High intensity developed land was absent in one site and emergent herbaceous wetland absent in seven sites. The average income measured at the 43 sites was $45,882.42 (US dollar). The range for income was $32,075.15 to $65,285.81 (all US dollar, Table 1).

**Table 1:** Mean, standard deviation, and range for land cover (percentage in 5 km buffer) and income (US dollar) used to examine bat-land cover/bat-income relationships

Variable	Mean	SD	Range
Developed, open space	6.82	4.38	2.02–22.27
Developed, low intensity	2.43	2.87	0.11–12.44
Developed, medium intensity	0.85	1.23	0.01–5.71
Developed high intensity	0.32	0.52	0–2.76
Barren land (rock/sand/clay)	0.39	0.93	0.01–5.77
Deciduous forest	32.82	28.89	0.01–87.51
Evergreen forest	12.00	9.15	0.59–45.05
Mixed forest	2.59	2.05	0.15–8.25
Shrub/scrub	5.93	5.13	0.52–21.04
Grassland/herbaceous	4.54	3.30	0.37–15.00
Pasture/hay	10.98	11.07	0.01–48.09
Cultivated crops	7.64	12.34	0.01–45.11
Woody wetlands	9.53	13.58	0.02–55.17
Emergent herbaceous wetlands	1.09	2.38	0–11.69
Median household income ($)	45,882.42	6539.29	32,075.15–65,285.81

**Fig. 1:**
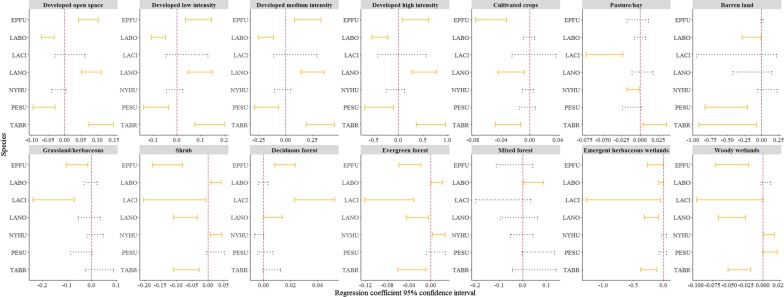
Generalized linear mixed model regression coefficient 95% confidence intervals (based on 1000 rounds of bootstrapping) in relation to 0 (0 as red vertical dash line; non-overlapping means significant in yellow solid line, overlapping means non-significant in blue dash line) for bat-land cover relationships; EPFU, *Eptesicus fuscus*; LABO, *Lasiurus borealis*; LACI, *L. cinereus*; LANO, *Lasionycteris noctivagans*; NYHU, *Nycticeius humeralis*; PESU, *Perimyotis subflavus*; TABR, *Tadarida brasiliensis*

For species-specific bat activity–land cover relationships, we found that *E. fuscus* activity was positively correlated to all four types of urban development as well as deciduous forest (Table 2, Fig. 1). Negative correlations were found between *E. fuscus* activity and evergreen forest, shrub, grassland/herbaceous, cultivated crops, and both types of wetlands (Table 2, Fig. 1). *Lasionycteris noctivagans* showed similar association patterns to *E. fuscus* except that *L. noctivagans* did not respond to grassland/herbaceous land cover. *Tadarida brasiliensis* had positive correlations with urban development, and the same negative bat activity–land cover relationships as *L. noctivagans*. Additionally, *T. brasiliensis* activity decreased as barren land cover increased (Table 2, Fig. 1).

**Table 2:** Generalized linear mixed regression model coefficient 95% confidence interval for bat-land cover, and bat-income relationships; significant relationships in italic

Variable	EPFU	LABO	LACI	LANO	NYHU	PESU	TABR
Developed open space	*0.0423 to 0.1030*	*− 0.0707 to − 0.0315*	− 0.0283 to 0.0615	*0.0520 to 0.1135*	− 0.0392 to 0.0049	*− 0.0958 to − 0.0290*	*0.0742 to 0.1493*
Developed low intensity	*0.0368 to 0.1449*	*− 0.1060 to − 0.0476*	− 0.0466 to 0.1315	*0.0491 to 0.1501*	− 0.0435 to 0.0262	*− 0.1392 to − 0.0332*	*0.0771 to 0.1999*
Developed medium intensity	*0.0869 to 0.3337*	*− 0.2495 to − 0.1107*	− 0.1079 to 0.2953	*0.1450 to 0.3602*	− 0.1014 to 0.0526	*− 0.2908 to − 0.0655*	*0.1949 to 0.4617*
Developed high intensity	*0.0821 to 0.6138*	*− 0.5413 to − 0.2088*	− 0.4174 to 0.5624	*0.2798 to 0.7725*	− 0.2450 to 0.1284	*− 0.6791 to − 0.0953*	*0.3627 to 0.9565*
Cultivated crops	*− 0.0763 to − 0.0324*	− 0.0086 to 0.0072	− 0.0249 to 0.0374	*− 0.0445 to − 0.0075*	− 0.0101 to 0.0058	*− 0.0145 to 0.0083*	*− 0.0484 to − 0.0125*
Pasture/hay	− 0.0179 to 0.0112	− 0.0074 to 0.0076	*− 0.0734 to − 0.0232*	− 0.0109 to 0.0179	*− 0.0178 to − 0.0008*	*− 0.0235 to 0.0017*	*0.0040 to 0.0362*
Barren land	− 0.0080 to 0.0231	*− 0.2831 to − 0.0099*	− 0.9462 to 0.2207	− 0.4192 to 0.1504	− 0.061 to 0.2311	*− 0.8276 to − 0.2072*	*− 0.9151 to − 0.0750*
Grassland/herbaceous	*− 0.1033 to − 0.0152*	− 0.0314 to 0.0232	*− 0.2376 to − 0.0713*	− 0.0546 to 0.0348	− 0.0179 to 0.0466	− 0.0846 to 0.0030	− 0.0253 to 0.0890
Shrub	*− 0.1740 to − 0.0801*	*0.0097 to 0.0412*	*− 0.2020 to − 0.0065*	*− 0.1086 to − 0.0334*	*0.0081 to 0.0441*	− 0.0039 to 0.0514	*− 0.1076 to − 0.0269*
Deciduous forest	*0.0085 to 0.0236*	− 0.0036 to 0.0034	*0.0227 to 0.0528*	*0.0003 to 0.0138*	− 0.0068 to 0.0002	− 0.0041 to 0.0073	− 0.0006 to 0.0127
Evergreen forest	*− 0.0588 to − 0.0178*	*0.0004 to 0.0221*	*− 0.1213 to − 0.0309*	*− 0.0447 to − 0.0045*	*0.0031 to 0.0268*	− 0.0073 to 0.0272	*− 0.0604 to − 0.0084*
Mixed forest	− 0.1124 to 0.0421	*0.0030 to 0.0879*	− 0.2002 to 0.0334	− 0.0946 to 0.0641	− 0.0513 to 0.0434	− 0.0027 to 0.1364	− 0.0429 to 0.1412
Emergent herbaceous wetlands	*− 0.2695 to − 0.0023*	*− 0.0775 to − 0.0007*	*− 1.2877 to − 0.0500*	*− 0.3173 to − 0.0799*	− 0.0263 to 0.0526	− 0.0734 to 0.0516	*− 0.3799 to − 0.1094*
Woody wetlands	*− 0.0706 to − 0.0209*	− 0.0028 to 0.0118	*− 0.0987 to − 0.0003*	*− 0.0663 to − 0.0259*	*0.0016 to 0.0169*	*0.0001 to 0.0214*	*− 0.0520 to − 0.0183*
Income	− 1.2980 to 0.9523	*0.1031 to 1.3445*	*− 3.1720 to − 0.4910*	− 1.3846 to 0.8113	*0.4060 to 1.8020*	− 1.2866 to 0.6646	− 0.8104 to 1.8686

EPFU, *Eptesicus fuscus*; LABO, *Lasiurus borealis*; LACI, *L. cinereus*; LANO, *Lasionycteris noctivagans*; NYHU, *Nycticeius humeralis*; PESU, *Perimyotis subflavus*; TABR, *Tadarida brasiliensis*

In contrast to the three species above, *L. borealis* and *P. subflavus*, had negative relationships with all 4 types of urban development land cover (Table 2, Fig. 1). Activity of both species also decreased with increased barren land cover. As evergreen forest, mixed forest, and/or shrub land cover increased, so did *L. borealis* activity. However, there was a negative relationship between emergent herbaceous wetland land cover and *L. borealis* activity. The only land cover type that increased *P. subflavus* activity was woody wetland (Table 2, Fig. 1). *Lasiurus cinereus* and *N. humeralis* activity was not associated with urban development land cover*. Lasiurus cinereus* activity increased with deciduous forest cover and decreased with evergreen forest, shrub, grassland/herbaceous, pasture/hay, and both types of wetland cover (Table 2, Fig. 1). *Nycticeius humeralis* activity decreased with pasture/hay cover and increased with evergreen forest, shrub, or woody wetland cover (Table 2, Fig. 1).

**Fig. 2:**
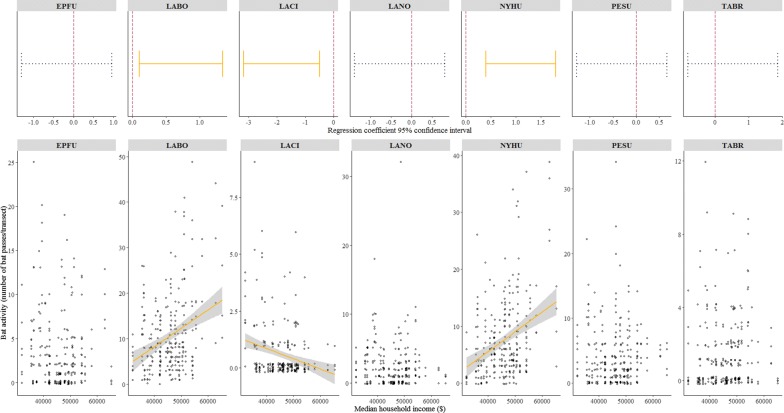
Generalized linear mixed model regression coefficient 95% confidence intervals (based on 1000 rounds of bootstrapping) in relation to 0 (0 as red vertical dash line; non-overlapping means significant in yellow solid line, overlapping means non-significant in blue dash line) and scatter plot with estimated significant regression trend line/range for bat-income relationships; EPFU, *Eptesicus fuscus*; LABO, *Lasiurus borealis*; LACI, *L. cinereus*; LANO, *Lasionycteris noctivagans*; NYHU, *Nycticeius humeralis*; PESU, *Perimyotis subflavus*; TABR, *Tadarida brasiliensis*

As hypothesized, there was a positive relationship between bat activity and income for *L. borealis* and *N. humeralis* (Fig. 2). We also found a negative correlation between income and *L. cinereus* activity (Fig. 2). No bat-income relationship was found for the other species. In the post hoc modeling for *L. borealis* and *N. humeralis*, we examined interactions between income and land cover type that had a positive relationship on bat activity. We found a positive interaction term between income and evergreen forest for *L. borealis* activity (Table 3), suggesting the positive effect of income on *L. borealis* activity was stronger in areas with more evergreen forest. No interaction term was found for other land cover–income pairs for *L. borealis* and *N. humeralis*. We examined interactions between income and land cover variables that had a negative relationship with *L. cinereus* activity. We found a positive interaction term between income and woody wetland for *L. cinereus* activity (Table 3), suggesting *L. cinereus* activity would decrease more as income increased in areas with more woody wetlands.

**Table 3:** Post hoc generalized linear mixed regression model coefficient 95% confidence interval for income and land cover interaction term for bat activity (significant terms in italic); other variables in the models were reported in Additional file 2

Species	Variable interaction term	Regression coefficient 95% confidence interval
*Lasiurus borealis*	*Income × evergreen forest*	*0.0044 to 0.1206*
	Income × mixed forest	− 0.2522 to 0.3651
	Income × shrub	− 0.1867 to 0.0051
*Lasiurus cinereus*	Income × evergreen forest	− 1.8770 to 1.3499
	Income × shrub	− 1.5448 to 2.0974
	Income × grassland/herbaceous	− 1.8732 to 0.9881
	Income × pasture/hay	− 2.1516 to 0.0587
	*Income × woody wetlands*	*0.2140 to 2.3580*
	Income × emergent herbaceous wetlands	− 0.4870 to 2.2350
*Nycticeius humeralis*	Income × evergreen forest	− 0.1348 to 0.0102
	Income × shrub	− 0.1485 to 0.0530
	Income × woody wetlands	− 0.2713 to 0.0537

## Discussion

Our study showed that bat-land cover associations at the landscape scale were consistent with previous studies at finer scales. Both *L. borealis* and *N. humeralis* are tree roosting species [37, 38] associated with tree cover at the local scale (e.g. [4, 39, 40]). Herein, we found the same relationships between bat activity and certain types of forest cover at a much broader scale. Moreover, *N. humeralis* can additionally roost in buildings whereas *L. borealis* roosts exclusively in trees [37, 38, 41]. Thus, it was not surprising that we found *L. borealis* negatively associated with urban development whereas the response of *N. humeralis* to urban development was neutral.

Interestingly, our study also showed that the luxury effect could be found across landscapes. After accounting for spatial and temporal relationships, two previously studied species, *L. borealis* and *N. humeralis*, showed the same positive response to affluence as found in a single-city study [4]. However, certain ecological traits might not be sufficient to explain why the luxury effect could be found across different landscapes beyond urban ecosystems. This is because income was not correlated with any land cover type across our study area. Furthermore, there was a positive interaction term between income and evergreen forest cover for *L. borealis*, suggesting that the luxury effect was more profound in areas with more evergreen forest. This interaction, consistent with Li and Wilkins [4], suggests that income and land cover habitat might function through different mechanisms to shape the bat activity patterns.

Certain mechanisms have been suggested for the luxury effect. For sedentary organisms, such as plants, human actions determined by affluence can directly alter the physical environment, such as water availability, which may favor certain ecological traits [1, 6, 7]. Similarly, water sources could potentially affect more mobile species. Our land cover data has a 30-m resolution [42], which did not capture small water bodies such as small creeks, ponds, or swimming pools. The importance of both natural and artificial water source to bats has been established (e.g. [43–46]) and wealthier people prefer to purchase properties that include water bodies for various reasons [27, 28, 47]. Thus, we speculate that water availability, especially small water bodies might contribute to the luxury effect found at the broad scale.

In addition to water sources, food or prey sources, which may be influenced by multiple environmental variables have been shown to affect predators’ distribution [48]. Studies of highly mobile meso-carnivores demonstrated that wealthier neighborhoods had higher vegetation coverage to support more small mammals, which were the prey for urban coyotes [14, 49]. Similarly, the importance of insect diversity and abundance to bats have been well documented (e.g. [50, 51]). Even though very few studies have compared insect diversity and abundance across landscapes with varying affluence, it is evident that high affluence organic farming promotes high insect diversity and abundance [23, 24, 52, 53]. Thus, we also speculate that insect food sources might also contribute to the luxury effect. Future studies should examine the luxury effect on insects at broad spatial scales.

Both water and food sources could potentially contribute to the luxury effect we found for *L. borealis* and *N. humeralis*. However, it is puzzling that we also found *L. cinereus* activity negatively associated with income, independent of land cover. In Li and Wilkins [4], *L. cinereus* activity was only recorded on the outskirts of the city, thus was not tested for the luxury effect. *Lasiurus cinereus* is a solitary tree foliage roosting species [54], which is consistent with its association with deciduous forests in our study. *Lasiurus cinereus* also prefers open space to forage [41, 54]. However, none of these ecological traits explains the association with low income areas. One possible explanation could be that *L. cinereus* responded to nighttime illumination. Regional economic activities have been found to be correlated to nighttime illumination [55]. How bats responded to nighttime illumination have been found for certain species (e.g. [56, 57]), a study on nighttime light effects on *L. cinereus* may help to understand our result.

As Ackley et al. [2] suggested, affluence might be a holistic index of habitat structure that characterizes a combination of many different aspects of the environment. Thus, future studies should focus on understanding what exactly bats perceive as habitat. It is also important to point out that the income range in this study was not particularly large. The mobile transect survey we used in our study required a certain length of a transect and a constant sampling speed [58] and thus prevented sampling in some urban areas with the highest income. Additionally, our study region lacked extreme low-income areas. Future studies should consider other sampling methods to include high-income urban areas and expand the spatial scale to include areas with lower oncome. Lastly, based on the intermediate disturbance theory, the relationship between bat activity and income could be non-linear [59, 60], which is also a topic for future studies.

For species that did not show linear association with income, our study provides a broad scale examination of their land cover associations, which are consistent with each species ecology based on fine scale studies. For example, *P. subflavus*, is tree roosting species that uses wetlands and forages on aquatic insects [44, 61–63]. Our analysis shows the same association with woody wetland at the landscape scale. Our results also support the importance of functional guilds in land cover selection for bats [36, 64–66]. For example, open space foragers, such as *E. fuscus* or *T. brasiliensis* showed associations with urban cover whereas tree roosting species generally associated with forest cover. Furthermore, our results showed that no single land cover type could benefit all seven species, suggesting the importance of conserving different land cover types for the conservation of bats. The species-specific bat activity-land cover associations also call for more fine scale ecological studies to answer questions such as why *L. noctivagans* showed a positive association with deciduous forests but a negative one with evergreen forest. Lastly, our study analyzed the composition of land cover types and it is important to note that the spatial configuration of land cover types also affects the distribution of organisms [67]. Future studies should address how connectivity (or fragmentation) of certain types of land cover affects bat activity.

## Conclusion

Our study demonstrated that the luxury effect in urban ecosystems can be found beyond cities. It is an ecological pattern that is evident across different landscapes at a broad spatial scale. The luxury effect may function through a mechanism or mechanisms different from simple bat-land cover associations. Fine scale studies are needed to better understand the specific habitat requirements of bats, especially due to the rapid urbanization and increase of anthropogenic environments. We also suggest that the luxury effect should be examined in different taxa, worldwide. The consistency of the luxury effect between spatial scales we found in the state of North Carolina, USA and in one city, Waco, Texas, USA, suggests that the luxury effect is broad and scalable. Importantly, the luxury effect has cascading impacts on both the environment and on humans [7]. There is an enormous value of bats to humans because of the ecosystem services they provide such as insect predation, seed dispersal, and pollination [68–71]. Potentially, the ecosystem services provided by bats may be unequally received by people with different levels of income, as found in urban vegetation, due to the luxury effect [3, 31, 72].

## Methods

### Study region and sampling site selection

**Fig. 3:**
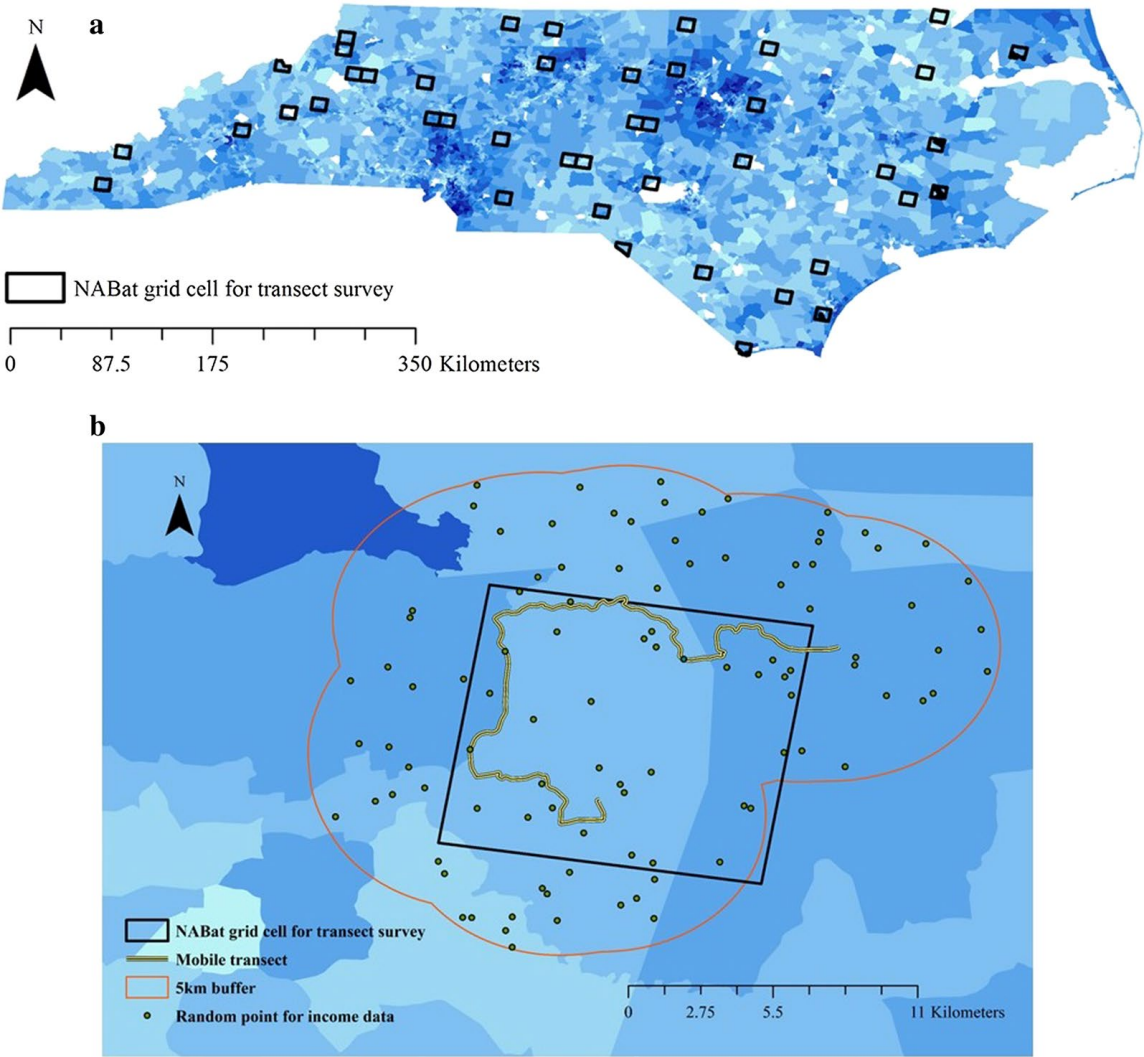
**a** North American Bat Monitoring Program (NABat) grid cells selected for the mobile transect survey across North Carolina with the 2016 American Community Survey 5-year estimates of median household income shown at census block group scale (darker shade in blue indicates higher level of income, white indicates no data, income data available at https://www.census.gov/programs-surveys/acs/data/data-via-ftp.html). **b** A demonstration of a survey site, including one NABat grid cell, one mobile transect, one 5 km buffer, and 100 random points for extracting income data

We recorded bat activity across North Carolina (NC), USA (Fig. 3a). All seven target species have statewide distributions [35]. North Carolina is approximately 140,000 km^2^ with three geographical regions (west to east): mountains, piedmont, and coastal plain. According to US Census data in 2016, the median household income of NC is approximately $50,000 (the 38th in the US), lower than the national value (approx. $59,000). The two largest metropolitan areas (Charlotte, NC and Raleigh, NC) are in the piedmont region. Smaller urban centers (e.g. Asheville, NC and Wilmington, NC) are present in the mountains or coastal plain regions, functioning as regional hubs and tourism destinations [73].

We followed the North American Bat Monitoring Program (NABat) grid cell framework to select sites for field sampling. Detailed NABat grid cell framework and sampling site selection criteria were described in Loeb et al. [58] and Li and Kalcounis-Rueppell [35]. The NABat grid cell framework divides North America into 10 km by 10 km grid cells. All grid cells are ranked with a generalized random-tessellation stratified survey design algorithm, which allows subsampling of NABat grid cells to form a set of sites that are spatially balanced yet randomized [58]. We treated one NABat grid cell as a sampling site. In 2015, we selected 32 sites for field sampling. In each year from 2016 to 2018 we re-evaluated site availability based on logistic constraints and added sites to replace sites that were no longer available for sampling. We had 39, 33, 34 sites for 2016 to 2018 field seasons respectively. Overall, we had 43 unique sampling sites across the state of NC that represented different landscapes (Fig. 3a). Within the 43 sties we collected 276 individual samples.

### Bat mobile transect survey and acoustic identification

To sample bat activity at a site, we used the NABat mobile transect survey protocol. At a sampling site, we mapped a 30–35 km non-overlapping transect before the field season. Details of transect road selection and an example of the transect spatial layout are presented in Li and Kalcounis-Rueppell [35]. Briefly, we used Anabat SD2 bat detectors and related car mount accessories (Titley Scientific, Australia) for the NABat mobile transect survey protocol to record bat acoustic activity. All sampling was in June and July of each year. We only sampled on nights with no rain or heavy fog, and with wind speeds less than 10 km/h. The transect survey started 45 min after sunset and was driven at a constant speed of 32 km/h and followed all local traffic rules. Each transect was driven twice a season within 7 nights. We also coordinated transect sampling dates at sites across years for consistency. For example, if a site was sampled early in June the first year, we endeavored to sample it early in June in subsequent years. For each transect sample, we recorded date, temperature, relative humidity, wind speed, and percent cloud cover at the beginning and the end of the survey. We used the mean of each variable collected at the beginning and the end of the survey (except for date) as survey covariates for the transect sample.

All acoustic recordings were processed with Analook (Version 4.2g, Titley Scientific, Australia) for manual species identification, as in Li and Kalcounis-Rueppell [35]. We only included recordings with at least three complete and clear bat echolocation pulses for species identification and defined each qualified recording as a bat pass. The first author compared all recordings based on bat echolocation pulse characteristics (measured by AnaLook) suggested by O’Farrell et al. [74] and Kunz and Parsons [75] with a known bat echolocation library described in Li and Kalcounis-Rueppell [35] to identify species. The species identification process was conservative. Certain species pairs, such as *E. fuscus* and *L. noctivagans*, or *L. borealis* and *N. humeralis*, can produce similar echolocation pulses. For these pairs, we did not identify a recording to species unless the unique characteristics of a species were found in multiple pulses. Further, we did not differentiate *L. borealis* from *L. seminolus* (Seminole bats) as these two species cannot be identified acoustically [76]. We only identified the seven target species. For each species, the dependent variable for statistical analyses was bat activity measured as number of bat passes per transect. We did not standardize bat activity by survey time because the amount of time to drive a transect was uniform (approximately 60 min). Further, all dependent variables and statistical analyses were species specific and we did not make any comparisons across species because there are species biases in acoustic methods of bat surveys [77].

### Land cover and affluence data collection

We used land cover as an indicator of general habitat structure experienced by bats [78, 79]. Our land cover data source was the National Land Cover Database 2016 (NLCD 2016, [42]). Using ArcMap (10.4.1, ESRI, California), we generated a 5 km radius buffer around each transect. We selected 5 km as the buffer radius because it is the common nightly active range of most target bat species [30, 35, 80, 81] and we were interested in examining the luxury effect at the landscape scale, not the local scale [40, 67, 82]. We used ArcMap to extract land cover raster images from NLCD 2016 and used FRAGSTATS [83] to calculate the percentage of each land cover type within the buffer as the indicator of the amount of each land cover type available. All land cover types described in Yang et al. [42] were considered. A preliminary multi-collinearity test of all land cover types showed that all four developed land cover types (open space, low, medium, high intensities) were highly correlated (all correlation coefficients > 0.6, all variance inflation factors > 5, Additional file 1; [84]).

To avoid multi-collinearity, we modeled each land cover type separately. Further, we opted to examine all land cover types without combining highly correlated land cover types because different types of urban land cover may function as specific habitat structure for bats. For example, high intensity developed land which includes urban center office buildings might function as roosting habitat for bats [85, 86], and open space developed land which includes parks might function as foraging habitat [87, 88].

We used median household income as the indicator of affluence [1, 7]. For our median household income variable, we used the 2016 American Community Survey (ACS) 5-year estimates published by the US Census (https://www.census.gov/programs-surveys/acs/data/data-via-ftp.html), because we needed income data from all areas regardless of the community size (Fig. 3a; [89]). Median household income was available at the census block group scale from the ACS 5-year estimates. Because most census blocks did not align with the 5 km buffer and some blocks only overlay with the buffer by a small portion, we decided to use random points to calculate the weighted average median household income for each buffer. At each bat sampling site, within the 5 km radius buffer of the transect, we used ArcGIS to generate 100 spatially random points (Fig. 3b) to extract 100 median household income measurements for a site. We explicitly excluded census block groups with no data during this process. We calculated the mean of 100 measurements and used the mean as the “income” variable to represent the affluence of each bat sampling transect. Lastly, we calculated the variance inflation factor for income with land cover and found no collinearity (all correlation coefficients < 0.3, all variance inflation factors < 2, Additional file 1).

### Statistical analyses

All analyses were done in R (version 3.4.2, [90]). We modeled each independent variable separately then explored possible interaction terms, post hoc, for selected land cover type and income pairs. We found that for all seven species, bat activity variance was different from the mean (all variance to mean ratios > 4). Therefore, we modeled bat activity with a negative binomial distribution [91, 92]. We also tested all possible spatial and temporal autocorrelations in bat activity in the preliminary analysis, and adjusted models accordingly [93].

Temporal autocorrelation of bat activity could occur between the two sample nights at a site within a season. Since all sites had two nights within a season, we first used Wilcoxon signed-rank test to compare all night one with all night two, independent from survey covariates. We found a difference between night one and night two (p < 0.05 for all species). Next, we used generalized linear models as the preliminary analysis to if the survey covariates would affect bat activity. We found that all species except for *L. noctivagans* were significantly affected by temperature (Additional file 2), consistent with previous work in the area [94]. Other survey covariates mentioned above had no effect on bat activity. Therefore, we included temperature as a covariate for all bat activity-land cover/income models except for *L. noctivagans*.

**Fig. 4:**
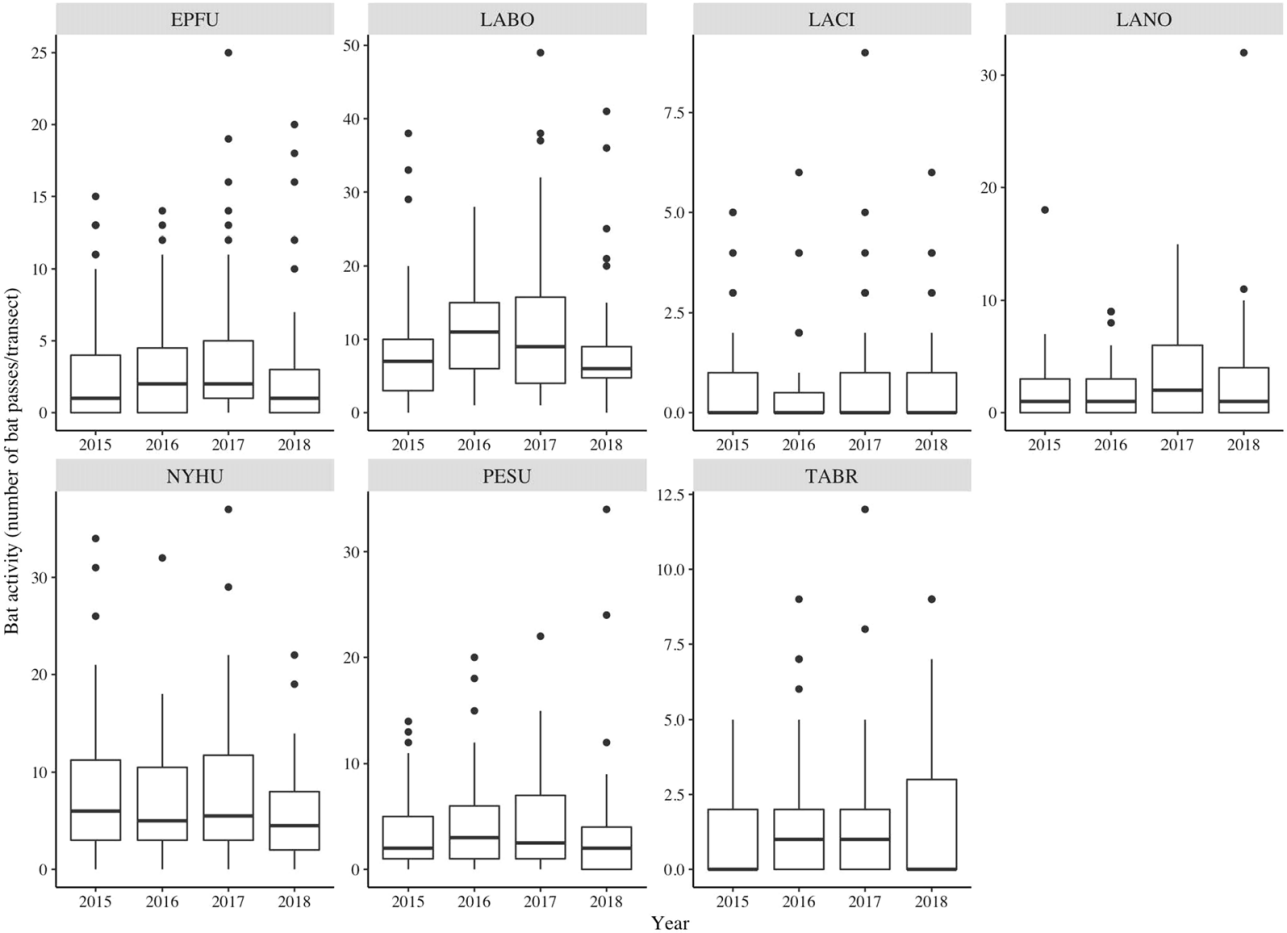
Box plot of species-specific bat activity (number of bat passes per transect) in relation to sampling years; all species responded to year significantly; EPFU, *Eptesicus fuscus*; LABO, *Lasiurus borealis*; LACI, *L. cinereus*; LANO, *Lasionycteris noctivagans*; NYHU, *Nycticeius humeralis*; PESU, *Perimyotis subflavus*; TABR, *Tadarida brasiliensis*

All target bat species may migrate regionally during the winter and cause local population size difference between summers [95]. Hence, we also conducted preliminary analyses with generalized linear models to evaluate if year had an effect on bat activity. We found that year was a significant variable for all bat species (Fig. 4, Additional file 2). Thus, we used generalized linear mixed-effects models to model bat activity-land cover/income relationship with year as a random effect using R package lme4 [84]. We further tested if the effect of temperature was nested within years by testing the interaction term between year and temperature. We found that for *E. fuscus*, *N. humeralis*, and *P. subflavus*, the effect of temperature varied between years (Additional file 2). Thus, for these three species, temperature was modeled as a covariate nested within the random effect of year. In this way, temperature was treated as a random slope and the relationship between bat activity and temperature was allowed to vary in slope with each year.

To test for potential spatial autocorrelation, we used the null generalized linear mixed-effects model including only temperature and year for all species but *L. noctivagans*, and only year for *L. noctivagans*, to calculate model residuals for Moran’s I test using R package ape v5.2 [96, 97]. Using p < 0.05 as the significance criterion, we found spatial autocorrelation in *E. fuscus*, *L. borealis*, *L. cinereus*, *L. noctivagans*, and *N. humeralis*. For these five species, we calculated a spatial autocovariate using the distance-band based neighbor scheme and neighbor weights of inverse distance with R package spdep v0.8-1 [14, 96, 98]. We included the spatial autocovariate to account for spatial autocorrelation in the models for these species. Although other modeling techniques can address spatial autocorrelation [96, 99] we used this approach to support the negative binomial distribution for our bat activity variable (see [100]).

For a more robust inference, we applied bootstrapping to the generalized linear mixed-effects models and generated confidence intervals for independent variables as suggested by Bates et al. [101]. We resampled 1000 times for each generalized linear mixed-effects model. As we modeled each independent variable separately, our models took approximately 6–7 degrees of freedom (one independent variable of interest, covariates temperature, spatial autocovariate, random effect year of 4 categories). Thus, our sample size of 276 was adequate for generalized linear mixed-effects models and bootstrapping [102]. From the bootstrapping process, we generated the 95% confidence interval for land cover and income. If the 95% confidence interval did not overlap with 0, the independent variable had a significant relationship with bat activity. For post hoc modeling, we applied the same modeling method to the interaction term between income and each significant land cover type variable for each species where income was significant. We only conducted post hoc modeling for land cover type variables that had the same direction of effect, positive or negative, as income. For example, if income was found positively correlated with a species’ activity, we conducted post hoc modeling for the interaction term between income and each land cover type that was also positively correlated to bat activity. In a post hoc model, income and a selected land cover type and their interaction term were included (Additional file 2).

## Supplementary information

**Additional file 1.** Independent variable (land cover and income) multi-collinearity analysis result. Correlation scatter plots on the lower left of the graph and correlation coefficient between paired independent variables on the upper right of the graph; land cover codes are—cls_21_5km: Developed open space; cls_22_5km: Developed low intensity; cls_23_5km: Developed medium intensity; cls_24_5km: Developed high intensity; cls_31_5km: Cultivated crops; cls_41_5km: Pasture/hay; cls_42_5km: Barren land; cls_43_5km: Grassland/herbaceous; cls_52_5km: Shrub; cls_71_5km: Deciduous forest; cls_81_5km: Evergreen forest; cls_82_5km: Mixed forest; cls_90_5km: Emergent herbaceous wetlands; cls_95_5km: Woody wetlands.

**Additional file 2.** Regression model results. Preliminary regression model results on the effects of temperature and year on worksheet 1; interaction model results on income and selected land cover on worksheet 2.

## Data Availability

The bat datasets generated during and/or analyzed during the current study are available at the North American Bat Monitoring Program repository (https://www.nabatmonitoring.org/). Complied bat/land cover/income data are available from the corresponding author on reasonable request.

## Abbreviations

ACS: American Community Survey
EPFU: big brown bat *Eptesicus fuscus*
LABO: Eastern red bat *Lasiurus borealis*
LACI: hoary bat *Lasiurus cinereus*
LANO: silver haired bat *Lasionycteris noctivagans*
NABat: North American Bat Monitoring Program
NC: North Carolina
NLCD: National Land Cover Database
NYHU: evening bat *Nycticeius humeralis*
PESU: tri-colored bat *Perimyotis subflavus*
TABR: Mexican free-tailed bat *Tadarida brasiliensis*
