# Supplementary figures and images for: The luxury effect beyond cities: bats respond to socioeconomic variation across landscapes
Source: BMC Ecol. 2019 Nov 1;19:46. doi: 10.1186/s12898-019-0262-8 (PMC6825354; doi:10.1186/s12898-019-0262-8)

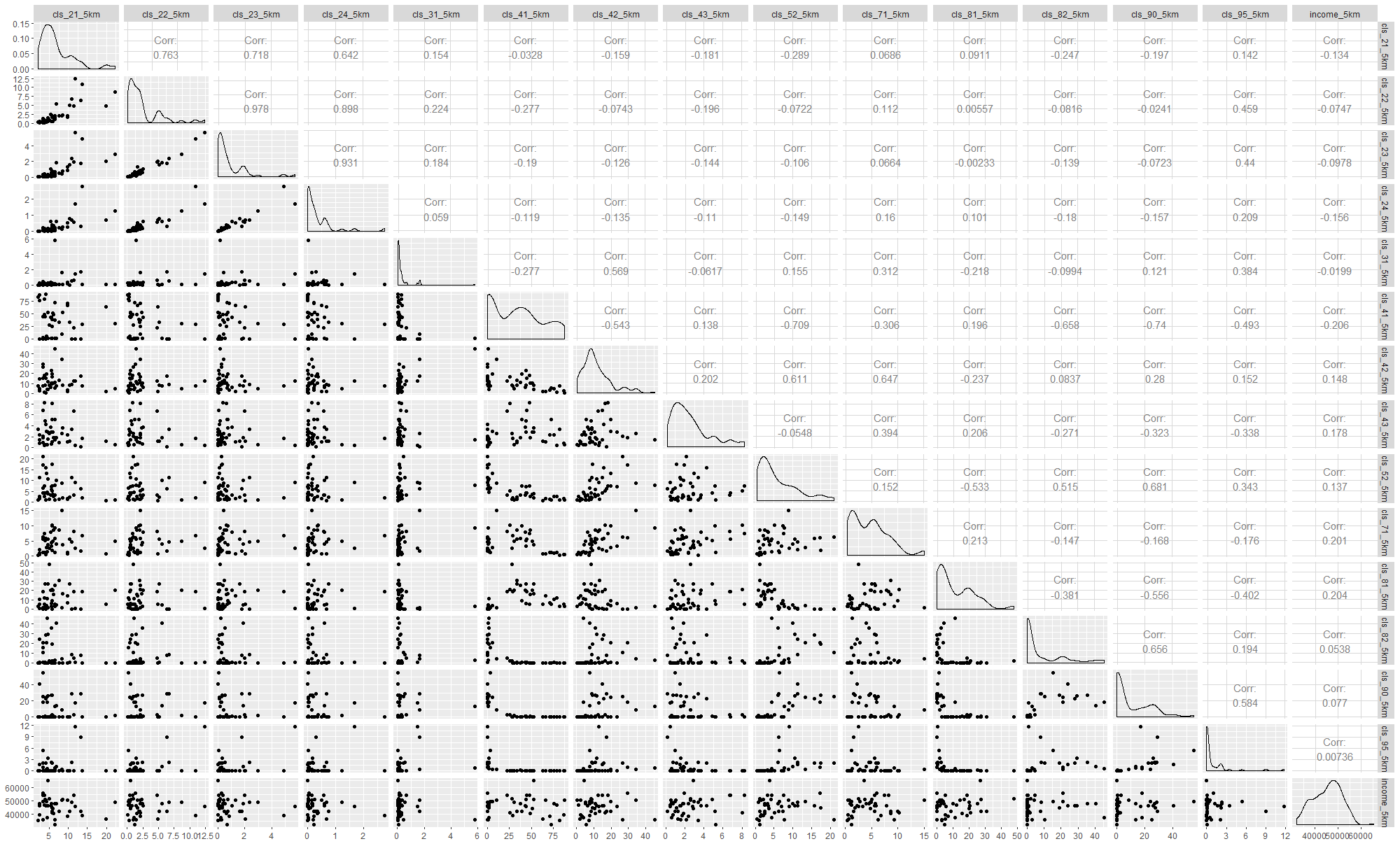

Supplement: Supplementary file 1 — Additional file 1. Independent variable (land cover and income) multi-collinearity analysis result. Correlation scatter plots on the lower left of the graph and correlation coefficient between paired independent variables on the upper right of the graph; land cover codes are—cls_21_5km: Developed open space; cls_22_5km: Developed low intensity; cls_23_5km: Developed medium intensity; cls_24_5km: Developed high intensity; cls_31_5km: Cultivated crops; cls_41_5km: Pasture/hay; cls_42_5km: Barren land; cls_43_5km: Grassland/herbaceous; cls_52_5km: Shrub; cls_71_5km: Deciduous forest; cls_81_5km: Evergreen forest; cls_82_5km: Mixed forest; cls_90_5km: Emergent herbaceous wetlands; cls_95_5km: Woody wetlands. [file 12898_2019_262_MOESM1_ESM.png]
